# Effect of β-1,3/1,6-glucan on gut microbiota of yellow-feathered broilers

**DOI:** 10.1186/s13568-022-01458-y

**Published:** 2022-09-06

**Authors:** Jingge Wang, Zibin Zheng, Hua Yang, Jie Chen, Yingping Xiao, Xiaofeng Ji, Zhenming Zhang, Hailian He, Baoan Ding, Biao Tang

**Affiliations:** 1 State Key Laboratory for Managing Biotic and Chemical Threats to the Quality and Safety of Agro-Products & Institute of Agro-Product Safety and Nutrition, Zhejiang Academy of Agricultural Sciences, Hangzhou, 310021 China; 2 College of Agriculture and Animal Husbandry, Qinghai University, Xining, 810016 China; 3 Hubei Key Laboratory of Animal Nutrition and Feed Science, Wuhan Polytechnic University, Wuhan, 430023 China; 4 Zhejiang Provincial Center for Animal Disease Prevention and Control, Hangzhou, 310020 China

**Keywords:** β-1,3/1,6-glucan, Yellow-feathered broilers, Gut microbiota, 16S rRNA

## Abstract

β-1,3/1,6-glucan as a prebiotic improves immune performance in animals. These functions are closely related to the effect of β-1,3/1,6-glucan on gut microbiota structure. However, the effect of β-1,3/1,6-glucan on the gut microbiota structure of broilers is unclear. The aim of this study was to confirm the effects of β-1,3/1,6-glucan on the cecal microflora structure of yellow-feathered broilers. This study monitored the antimicrobial resistance (AMR) level of *Escherichia coli* in feces of yellow-feathered broilers by standard broth dilution method and mastered the AMR level of chickens selected. The effects of β-1,3/1,6-glucan on gut microbiota were investigated by 16S rRNA sequencing. The results showed that the number of isolated multidrug-resistant *E. coli* strains accounted for 98.41%. At 14, 21, and 28 days of age, supplemented of 0.2%, 0.1%, and 0.1% β-1,3/1,6-glucan in yellow-feathered broiler diets significantly altered gut microbial composition, and beneficial bacteria *Alistipes*, *Bacteroides* and *Faecalibacterium* were significantly increased. These findings provide guidance and recommendations for β-1,3/1,6-glucan as a broiler feed additive to improve the growth of broilers.

## Introduction

Antibiotics used in animal breeding are one of the important sources of environmental antibiotics pollution. The increasing concentration of antibiotics in the environment makes bacteria evolve more extensive antimicrobial resistance (AMR) and further changes the composition of the microbial community (Danner et al. 2019). Antibiotics in livestock and poultry feeding processes can cause gut microbiota disorder and hinder animals’ average growth and metabolism (Lillehoj et al. 2018). The European Union, the United States and China imposed a complete ban of all anti-biotics in animal feed to promote growth in January 2006, January 2017 and October 2020, respectively (Salim et al. 2018). In promoting non-antibiotic breeding, finding suitable antibiotic substitutes is an essential link in the feeding mode of reducing the use of antibiotics. Currently, a number of possible alternatives to AGP are used, including acidifiers, probiotics, enzymes, algae and herbal products, microflora enhancers, and immuno-modulators (Salim et al. 2018; Seidavi et al. 2021).

β-glucan is considered a natural prebiotic with various biological functions, such as antioxidant, free radical scavenging, anti-tumor, anti-cancer, and immune activation, competing with pathogenic microorganisms for binding sites in intestinal epithelial cells to prevent inflammation (Baldassano et al. 2017; De Marco et al. 2021; Mo et al. 2017; Virginio et al. 2021; Xu et al. 2009). The main chain of β-1,3/1,6-glucan is a linear skeleton with β-1,3 bonds, and the side chain is a highly branched β-1,6 bond (Baldassano et al. 2017). It is commonly found in the cell walls of yeast, bacteria, fungi, algae, and plants (Baldassano et al. 2017). β-1,3/1,6-glucan was proved to be a source of substances with immune-stimulating properties in yeast cells in 1955 (Louis et al. 1955). It is a green, healthy, safe, and effective immune adjuvant (Bobadilla et al. 2013; Wu et al. 2016). β-1,3/1,6-glucan can reduce the colonization of *Salmonella* in the intestinal tract, relieve the level of intestinal and visceral organ injury caused by *Salmonella* infection, promote the number of probiotics such as *Bifidobacterium* and *Lactobacillus*, and also have a significant immune effect against parasitic and viral diseases (Horst et al. 2019; Shao et al. 2016). β-1,3/1,6-glucan is an essential component of a prebiotic-rich diet that promotes the growth and metabolism of the gastrointestinal microbiota (Horst et al. 2019; Shao et al. 2016).

Gut microbiota is a vast microbial ecosystem and complex ecosystem, easily affected by many factors, such as the environment, age, diet, and feed additive (Chen et al. 2020; Fassarella et al. 2021). Gut microbiota participate in vital physiological processes, such as energy homeostasis, metabolism, intestinal epithelial health, immune activity and neural development (Barko et al. 2018). Jayachandran et al. reported that the gut microbiota might realize the immunomodulatory effect of β-glucan as the mediator of the immune response (Jayachandran et al. 2018). At present, the research on β-glucan and gut microbiota mainly include rats (Aoe et al. 2019), mice (Shi et al. 2020), fish (Harris et al. 2020), dogs (Van et al. 2020), weaned piglets (Metzler-Zebeli et al. 2011), and calves (Virginio et al. 2021), which have not been reported in broilers. Among them, β-glucan distinctly raised the number of *Lactobacilli* and *Bifidobacteria* in the colon of weaned pigs, and also increased the concentration of butyrate in the stomach, cecum and colon, which may be beneficial to the intestinal development of weaned pigs (Metzler-Zebeli et al. 2011). β-glucan significantly increased acetic acid and butyrate concentrations in cecum of rats. The abundance of *Bacteroidetes* in cecum was significantly increased, and the abundance of *Firmicutes* in cecum was significantly decreased, which was helpful to induce secreted IgA to neutralize the toxins produced by microorganisms (Aoe et al. 2019).

In this study, the AMR of yellow-feathered broilers was monitored. The effects of β-1,3/1,6-glucan on the gut microbiota of broilers from hatching to 28 days of age were studied. This experiment analyzed the effect of β-1,3/1,6-glucan as a substitute antibody product in improving the gut microbial composition by 16S rRNA.

## Materials and methods

### Animals and sampling

This study was conducted on a farm in Huangzhong County, Qinghai Province. 240 1-day-old yellow-feathered broilers (male) with similar genetic and growth status were randomly divided into four groups with four replicates per group and 15 broilers per replicate. The broilers were housed in 16 cages with a size of 150 cm × 80 cm × 38 cm (15 broilers each). The negative control group (Y) was fed a basal diet. In the antibiotic group (T), 0.02% tylosin (Ringpu, China, with a purity of 10%) was added to the basal diet. β-1,3/1,6-glucan groups 1 and 2 (G1, G2) were supplemented with 0.1% and 0.2% β-1,3/1,6-glucan (Xingzhongcheng, China, from yeast) in basal diet, respectively. The composition and nutritional level of the basal diet are shown in Table 1.

**Table 1:** Ingredient composition and analysed nutrient contents of the basal diet

Composition	Proportion%		Nutritional level
Corn	56.81	Dry matter	84.36%
Soybean meal	28.00	Metabolic energy	11.84 (MJ/kg)
Soybean oil	2.00	Crude protein	19.55%
Wheat bran	5.30	Crude fat	4.47%
Yeast powder	3.00	Crude fiber	3.81%
Stone powder	1.96	Ash content	2.80%
Calcium hydrogen phosphate	1.10	Calcium	1.09%
Sodium Chloride	0.30	Phosphorus	0.64%
Methionine	0.23	Methionine	0.55%
Lysine	0.35	Lysine	1.45%
Threonine	0.08	Threonine	0.86%
Sodium sulphate	0.10		
Broiler multi mineral	0.12		
50% choline chloride	0.10		
Baking soda	0.05		
Premix	0.50		
Total	100.00		

Multi mineral content per kilogram of broiler: Copper 8 mg, Iron 60 mg, zinc 60 mg, manganese 60 mg, Selenium 0.15 mg and Iodine 0.35 mg. Nutritional levels are calculated values

The broilers had free access to feed and water during the experiment. We used incandescent lamps, which were slightly higher at 40 lx for the first week. After the second week, the light intensity gradually decreased and was 25 lx. The light was 24 h a day in the first week, then decreased to 0.5 h a day until the 26th day, and then kept for 17 h a day. The room temperature is controlled to be 35–33 ℃ on the 1st–7th day, 32.5–29.5 ℃ on the 8th–14th day, and 29–24 ℃ on the 15th–28th day. The humidity of the chicken house shall be kept between 60–70%, and the chicken house shall be disinfected and cleaned regularly according to the routine immunization procedure. Use 0.1% bromo-geramine solution to sterilize chickens, once a week, spray steriliza-tion. One yellow-feathered broiler was randomly selected from each replicate of each group for fresh feces collection at 7, 14, 21, and 28 days of age, respectively. One yellow-feathered broiler was randomly selected from each replicate of each group at 14, 21, and 28 days of age. After the chicks were dissected, the cecal contents were collected with sterile cotton swabs and put into a 5 ml sterile centrifuge tube. Refrigerate in a liquid nitrogen tank after sealing.

### Identification of Escherichia coli isolates and antimicrobial susceptibility testing

A total of 64 fresh fecal samples were collected at 7, 14, 21, and 28 days of age using Cary-Blair transport medium to isolate of *Escherichia coli*. Matrix-assisted laser desorption ionization-time-of-flight mass spectrometry (Bruker MALDI Biotyper System, Germany) was used for strain identification. AMR of *E. coli* strains was detected by the broth dilution method. The isolation and identification of *E. coli* strains and AMR detection methods were described in previous studies (Tang et al. 2021).

### DNA extraction, sequencing, and data analysis

According to the manufacturer’s instructions, the microbial genomic DNA was extracted from cecum contents of yellow-feathered broilers using the QIAamp DNA Stool Mini Kit (QIAGEN, US). The concentration of DNA in the extracted samples was detected with a NanoDrop 2000 spectrophotometer (ThermoFisher, US). The extracted DNA samples above were submitted to Shanghai Majorbio Bio-pharm Technology Co, Ltd for sequence analysis. The V3–V4 region of the bacterial 16S rRNA gene was amplified from each genomic DNA sample by using the primers 338F (5′-ACT CCT ACG GGA GCA GCA-3′)—806R (5′-GGA CTA CHV GGG TWT CTA ATT-3′). Sequencing libraries were then constructed using TruSeqTM DNA Sample Prep Kit and sequenced on an Illumina MiSeq 300 platform. After sample splitting of PE reads obtained by MiSeq sequencing, double-ended reads were firstly controlled and filtered according to sequencing quality and, at the same time, spliced according to the overlapping relationship between double-ended reads to obtain optimized data after quality control spliced. Then, sequence denoising methods (DADA2/Deblur) were used to process the optimized data, and Amplicon Sequence Variant (ASV) was used to represent the sequence and abundance information.

### Statistical analysis and visualization

All statistical analyses were performed by SPSS 23.0 (IBM, US) using an unpaired two-tailed Student’s t-test. Data are presented as the mean ± SEM. Results were considered significant when *P* < 0.05. Based on the representative sequences and abundance information of ASV, taxonomic analysis, community diversity analysis, species difference analysis, correlation analysis, and a series of statistical or visual analyses can be carried out. Alpha diversity indices (Chao and Shannon) via QIIME software (Version 1.7.0) and shown with R software (Version 2.15.3) (Lawley & Tannock 2017). Principal coordinate analysis (PCoA) was performed to analyze the beta diversity. Circos and Pie charts were generated to show taxa distribution at the phylum and genus levels.

## Results

### Isolation of E. coli and antimicrobial susceptibility

**Fig. 1:**
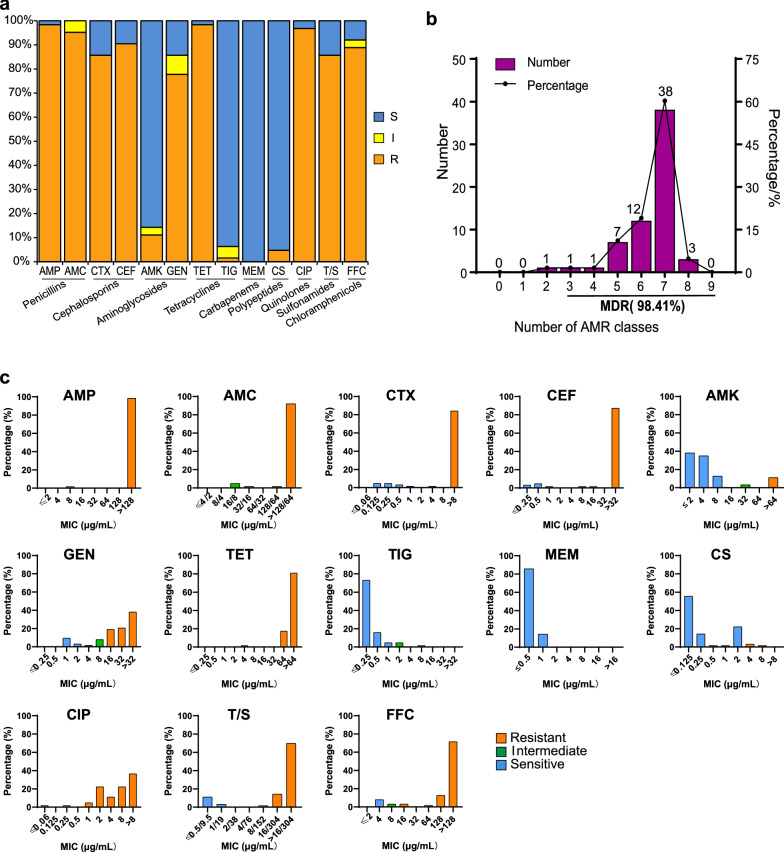
The susceptibility test results of 63 strains of *E. coli* to 13 antibiotics. **a** AMR rates of *Escherichia coli*. **b** The distribution of multidrug-resistant strains. **c** MIC distributions of 13 antibiotics in isolated *E. coli* strains. *MIC* minimum inhibitory concentration, *MDR* multidrug resistance, *AMP* ampicillin, *AMC* amoxicillin/clavulanate potassium, *CTX* cefotaxime, *CEF* ceftiofur, *AMK* amikacin, *GEN* gentamicin, *TET* tetracycline, *TIG* tigecycline, *MEM* meropenem, *CS* colistin, *CIP* ciprofloxacin, *T/S* sulfamethoxazole, *FFC* florfenicol

63 strains of *E*. *coli* were isolated and identified from 64 stool samples collected in this research, with an isolation rate of 98.44%. 63 of the *E*. *coli* strains to 13 antibiotics are shown in Fig. 1a and Fig. 1b. In terms of the MIC distribution (Fig. 1c), the MIC values of the antibiotics ampicillin (AMP), amoxicillin/clavulanate potassium (AMC), cefotaxime (CTX), ceftiofur (CEF), gentamicin (GEN), tetracycline (TET), ciprofloxacin (CIP), sulfamethoxazole (T/S) and florfenicol (FFC) were highly resistant to these antibiotics. AMP, AMC, CEF, TET, and CIP showed the highest AMR rates of over 90%, followed by CTX, T/S, and FFC, at 85.71%, 85.71%, 88.89%, while GEN exhibited AMR rates greater than 70%. Among the isolates, strains with multidrug resistance (MDR) accounted for 98.41% of the total isolates. In addition, their AMR rates for amikacin (AMK), colistin (CS) and tigecycline (TIG) were 11.11%, 4.76% and 1.59%, respectively. All tested *E*. *coli* strains were sensitive to meropenem (MEM). These results indicate that the AMR of chicken-derived *E*. *coli* in this region is profound.

**Fig. 2:**
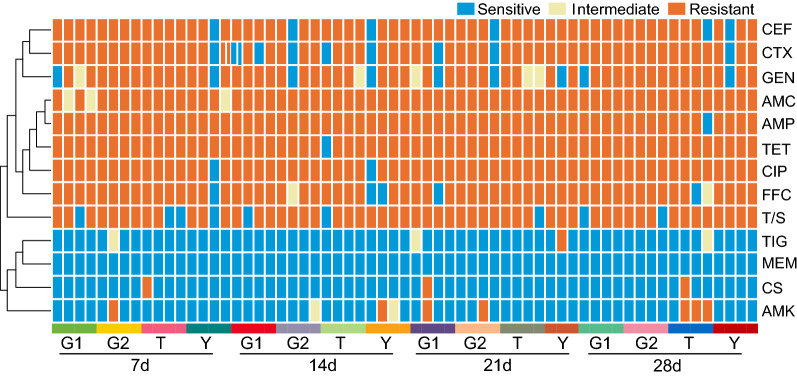
AMR profiles of 63 *E. coli* strains. *AMP* ampicillin, *AMC* amoxicillin/clavulanate potassium, *CTX* cefotaxime, *CEF* ceftiofur, *AMK* amikacin, *GEN* gentamicin, *TET* tetracycline, *TIG* tigecycline, *MEM* meropenem, *CS* colistin, *CIP* ciprofloxacin, *T/S* sulfamethoxazole, *FFC* florfenicol

As shown in Fig. 2 and Table 2, there were 18 types of AMR spectrum of 63 strains. AMP-AMC-CTX-GEN-CEF-CIP-T/S-TET-FFC was the most AMR phenotype of 31 strains (49.21%). There were 3 strains (4.76%) of 8 classes of antibiotics, 38 strains (60.32%) of 7 classes of antibiotics, 12 strains (19.05%) of 6 classes of antibiotics, 7 strains (11.11%) of 5 classes of antibiotics. It shows that the MDR of *E. coli* in this chicken farm is numerous, and there are many kinds of AMR profiles.

**Table 2:** The AMR patterns of 63 of *E. coli* strains

ID	Antibiotic-resistant pattern	Number	Percentage
1	AMP-AMC-CTX-GEN-CEF-CIP-T/S-TET-FFC	31	49.21
2	AMP-AMC-CTX-CEF-CIP-T/S-TET-FFC	4	6.35
3	AMP-AMC-CTX-GEN-CEF-CIP-TET-FFC	4	6.35
4	AMP-AMC-CTX-CEF-CIP-TET-FFC	3	4.76
5	AMP-CTX-GEN-CEF-CIP-T/S-TET-FFC	3	4.76
6	AMP-AMC-GEN-CEF-CIP-T/S-TET-FFC	2	3.17
7	AMP-AMC-CTX-AMK-GEN-CEF-CIP-T/S-TET	2	3.17
8	AMP-AMC-CTX-AMK-GEN-CEF-CIP-T/S-TET-FFC	2	3.17
9	AMP-AMC-CTX-AMK-GEN-CS-CEF-CIP-T/S-TET-FFC	2	3.17
10	AMP-AMC-CIP-T/S-TET-FFC	2	3.17
11	AMP-AMC-CTX-CEF-CIP-T/S-TET-TIG-FFC	1	1.59
12	AMP-AMC-CTX-GEN-CS-CEF-CIP-T/S-TET-FFC	1	1.59
13	AMP-AMC-GEN-CEF-CIP-FFC	1	1.59
14	AMC-CTX-AMK-GEN-CIP-T/S-TET	1	1.59
15	AMP-AMC-CEF-CIP-T/S-TET	1	1.59
16	AMP-AMC-CIP-T/S-TET	1	1.59
17	AMP-AMC-T/S-TET	1	1.59
18	AMP-AMC-TET	1	1.59

### 16S amplicon sequencing

**Fig. 3:**
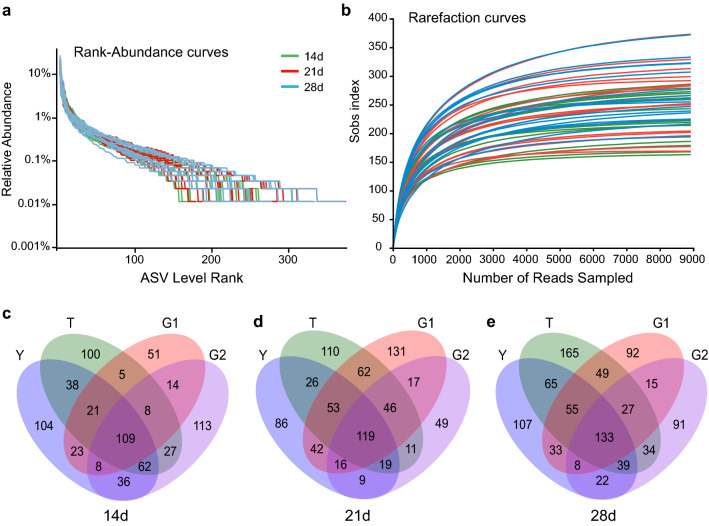
Analysis of ASV level data by amplification sequencing. ASV level Rank-Abundance curves **a**, Sobs exponential dilution curves **b**, and Ven diagram **c**, **d**, **e** of samples. The richness of species in the horizontal direction of the Rank-Abundance curve is reflected by the width of the curve, and the smoothness of the curve reflects the evenness of species in the sample. Different colors represent different grouped samples. The sobs index rarefaction curve was constructed to reflect the microbial diversity of each sample at different sequencing quantities. Different colors rep-resent different samples. Venn diagram can intuitively represent the similarity and overlap of ASV components of samples. Different colors represent different grouped samples

2,500,002 optimized 16S amplicon sequences with 1,034,571,892 bp and an average length of 414 bp were obtained from 48 cecal content samples. As shown in Fig. 3a, gentle rank-abundance curves at the ASV level indicate that species are evenly distributed in the sample community. As shown in Fig. 3b, the dilution curve under the sobs index of ASV level reflects that the amount of sequencing data of the submitted samples are reasonable. The coverage index of all sequencing samples was more significant than 0.99, indicating that the sequencing results were reliable. As shown in Venn Fig. 3c, d, e, the number of ASVs shared by the four groups at 14 days of age, 21 days of age, and 28 days of age were 109, 119, and 133, respectively, indicating that the bacterial species similarity in cecal contents of yellow-feathered broilers increased, possibly due to the gradual stability of gut microbiota with the increase of age.

### β-1,3/1,6-glucan administration altered broilers gut microbiota

**Fig. 4:**
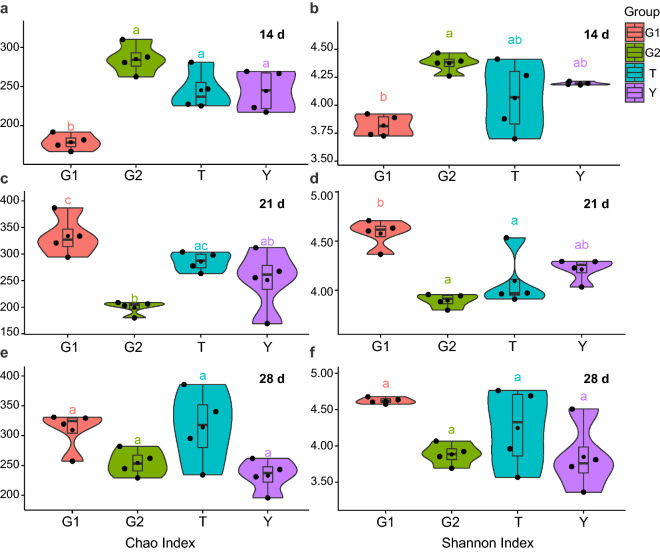
Alpha diversity, including Chao index **a**, **c**, **e** and Shannon index **b**, **d**, **f** in the control group (Y), antibiotic group (T), 0.1% β-1,3/1,6-glucan (G1), and 0.2% β-1,3/1,6-glucan (G2) groups in the cecum. Data was processed through log10, expressed as mean ± SEM (n = 8), and analyzed by one-way ANOVA analysis. The different superscript letters on the boxplot represent a significant difference *(P* < 0.05)

**Fig. 5:**
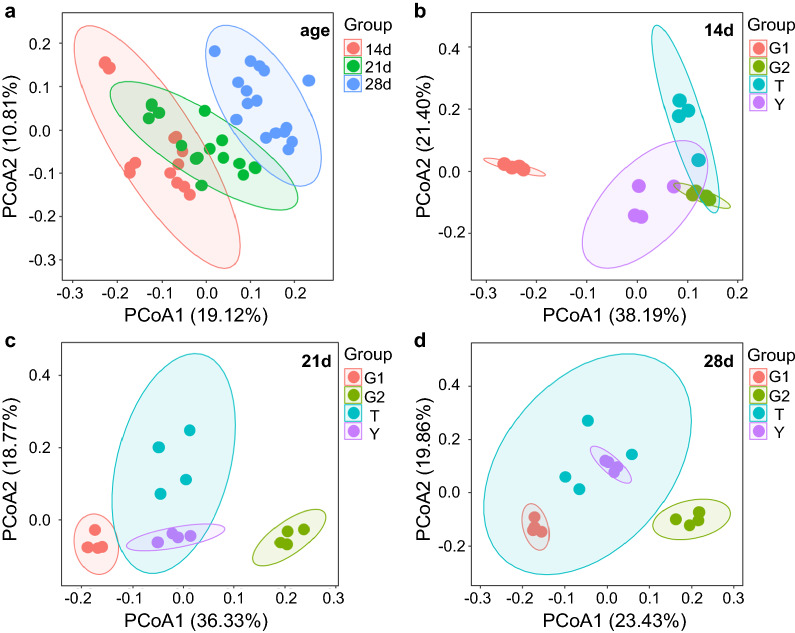
PCoA of the broilers gut microbial community composition of different age **a**, and the Y (control group), T (antibiotic group), G1 (0.1% β-1,3/1,6-glucan), and G2 (0.2% β-1,3/1,6-glucan) group of broilers aged 14 days **b**, 21 days **c** and 28 days **d** based on the Bray–Curtis distances showed distinct clusters. The cecum microbial community structure between the 14, 21, and 28 days of age were differentiated by colors (red, green, blue, respectively). The cecum microbial community structure between the Y, T, G1, and G2 groups were differentiated by colors (purple, blue, green, red, respectively)

The alpha-diversity indicated that the Chao index had a downward trend from the control group to the G1 group in 14 days (*P* < 0.05) (Fig. 4a, b), and there was an upward trend from 21 days (*P* < 0.05) (Fig. 4c, d) in yellow-feathered broilers cecum. The beta-diversity showed significant differences in cecal contents microbiota community at 14, 21, and 28 days of age (Fig. 5a). The beta-diversity showed significant changes in the microbial community members of cecal contents from the control group to T, G1, and G2 groups (Fig. 5b, c, d). Especially in the G1 group, its clustering is far from all other groups. In broilers cecum, T groups were closer to the control group. In 21 days, the G2 group was as far away from the control group as the G1 group.

**Fig. 6:**
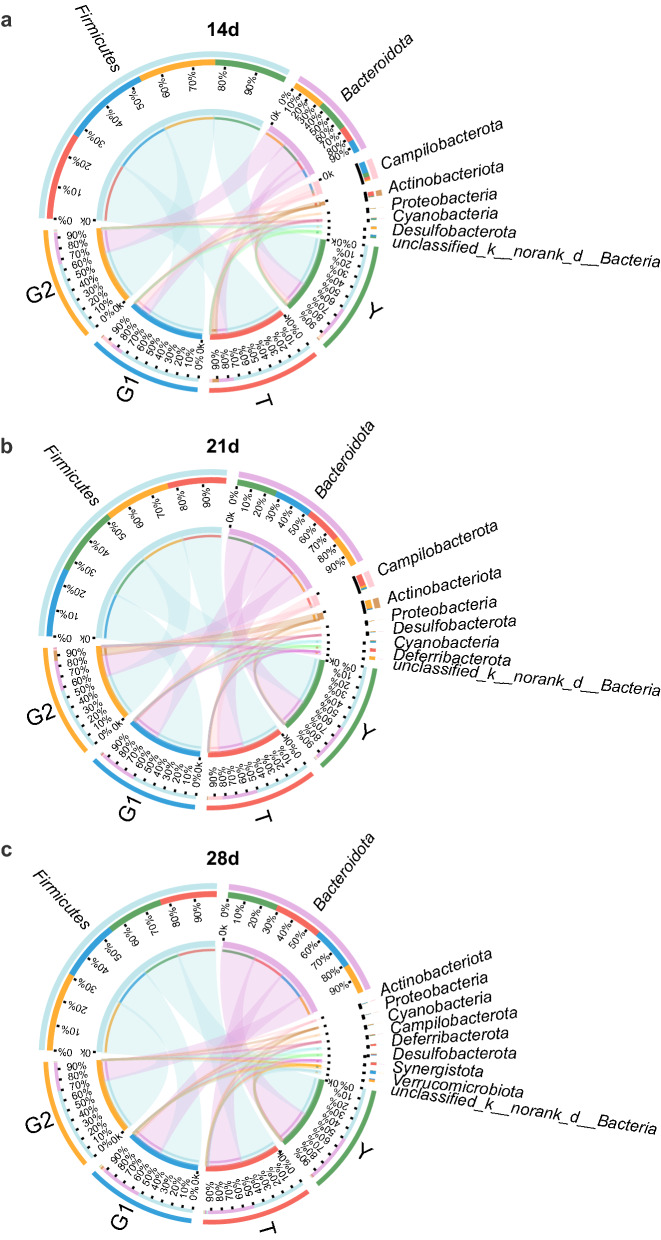
Bacterial communities in the cecum of broilers aged 14 days **a**, 21 days **b** and 28 days **c** at phylum level in groups Y (control group), T (antibiotic group), G1 (0.1% β-1,3/1,6-glucan), and G2 (0.2% β-1,3/1,6-glucan). The cecum microbial community structure between the Y, T, and G1, and G2 groups were differentiated by colors (green, red, blue, orange, respectively)

Next, we analyzed broilers’ microbiota composition in the cecum of broilers in the Y, T, G1, and G2 groups. Taxonomic analysis showed that the dominant bacteria phyla were *Firmicutes* and *Bacteriodetes*, accounting for more than 93.43% of the total sequences in all samples (Fig. 6). Compared to the control group, the relative abundance of *Firmicutes* in the T group and G1 group were increased by 13.64% (*P* < 0.05) and 12.60% (*P* < 0.05) in 14 days of broilers, respectively (Fig. 6a). However, the relative abundance of *Bacteriodetes* in the T group and G1 group was decreased by 14.47% (*P* < 0.05) and 16.94% (*P* < 0.05) in 21 days samples relative to the control group, respectively (Fig. 6b). *Firmicutes* were less by 20.17%, and *Bacteriodetes* increased by 18.65%, abundant in 28 days samples of the G2 group compared to the control group (*P* < 0.05) (Fig. 6c).

**Fig. 7:**
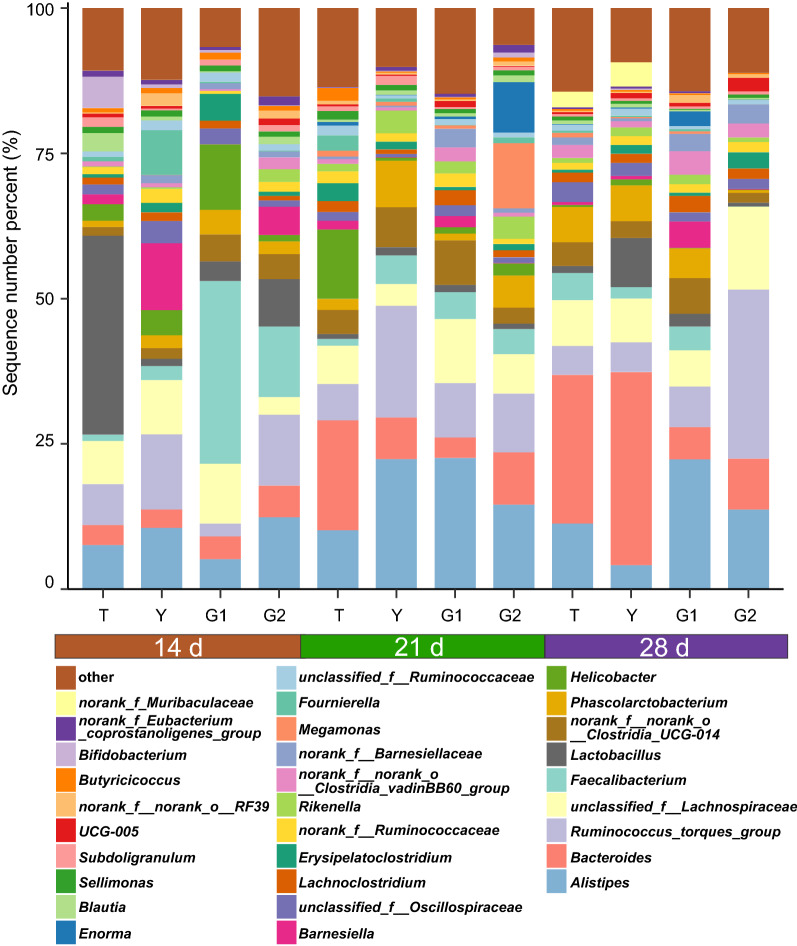
The top 30 features in Y (control group), T (antibiotic group), G1 (0.1% β-1,3/1,6-glucan), and G2 (0.2% β-1,3/1,6-glucan) groups of cecum microbiome in broilers. Each color indicates the relative abundance of a bacterial taxon on the bar chart

At the genus level, cecal contents samples were dominated by *Alistipes* (13.07%), *Bacteroides* (10.66%), *Ruminococcus*_torques_group (10.46%), unclassified_f__*Lachnospiraceae* (7.86%), *Faecalibacterium* (6.13%), and *Lactobacillus* (5.31%) (Fig. 7). Among the top 5 taxa, at 14 days of age, compared with the control group, *Faecalibacterium* had a downward trend in the T group, and it significantly rose in the G1 and G2 groups (*P* < 0.05). unclassified_f__*Lachnospiraceae* had an extremely significant decline in the G2 group compared with the control group at 14 days of age (*P* < 0.05). At the 21 days of age, the T group had a significant increase in *Bacteroides* and unclassified_f__*Lachnospiraceae* (*P* < 0.05), and the latter significantly rose in G1 and G2 group (*P* < 0.05) compared with the control group. At the 21 days of age, the T group had a significant decline in *Alistipes* (*P* < 0.05), *Ruminococcus*_torques_group (*P* < 0.05), and *Faecalibacterium* (*P* < 0.05), and *Ruminococcus*_torques_group significantly decline in G1 group (*P* < 0.05) compared with the control group. At 28 days of age, compared with the control group, the contents of *Alistipes* and *Bacteroides* in the G1 group were significantly increased and decreased (*P* < 0.05). At 28 days of age, compared with the control group, the G2 group had a significant increase in *Ruminococcus*_torques_group and unclassified_f__*Lachnospiraceae* and it significantly declines in *Bacteroides* (*P* < 0.05).

### Bacterial taxa differentially represented in broilers cecum microbiota

**Fig. 8:**
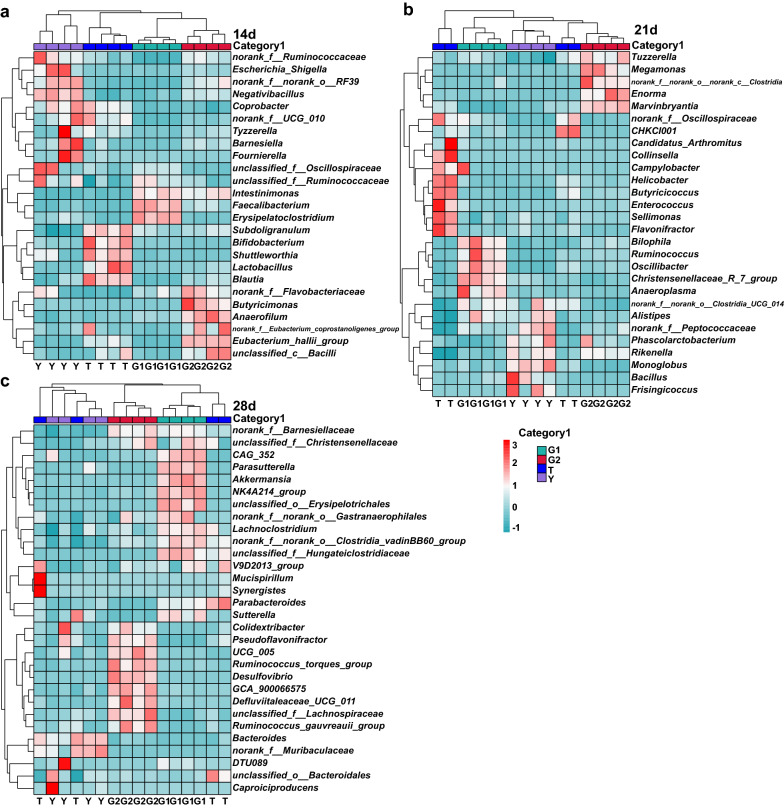
Hierarchically clustered heat map for the significantly different bacterial genera in cecal contents of 14 day old broiler **a**, 21 day old broilers **b** and 28 day old broilers **c**. Heat map indicated 83 bacterial taxa were identified by LEfSe (LDA > 3) in broilers at 14 days of age (n = 25) **a**, 21 days of age (n = 28) **b** and 28 days of age (n = 30) **c** cecum microbiome. The top 1000 features were used for lefse analysis. Heat map shows the average relative abundances on a Z-score

Broiler cecum bacterial features were analyzed by using lefse. Hierarchically clustered heat map for the significantly different bacterial genera in cecal contents of 14 day old broiler, 21 day old broilers and 28 day old broilers (Fig. 8). The abundance of these significantly different features was shown on the heat map. At 14 days of age (Fig. 8a), *Bifidobacterium* and *Lactobacillus* significantly increased in the T group compared with other groups (Y, G1, G2). At 21 days of age (Fig. 8b), *Megamonas* and *Enorma* had a significant increase in the G2 group compared with other groups (Y, T, G1), *Bacteroides*, and *Helicobacter* had a significant increase in the T group compared with other groups (Y, G1, G2). At 28 days of age (Fig. 8c), *Ruminococcus*_torques_group, *Desulfovibrio*, *Defluviitaleaceae_UCG_011*, and unclassified_f__*Lachnospiraceae* in the G2 group were significantly higher than other groups (T, Y, G1).

## Discussion

β-1,3/1,6-glucan can improve the immune level of the body, increase the expression of immune factors in intestinal and other damaged parts, and alleviate the damage to the body after pathogen infection (Bobadilla et al. 2013; Wu et al. 2016). The β-glucan regulating gut microbiota is a potential method to reduce disease susceptibility and improve growth performance in piglets (Luo et al. 2019). In this study, we detected the AMR of yellow-feathered broilers to 13 antibiotics from birth to day 28 and found that broilers in this study were highly resistant to multiple antibiotics. There was no difference in AMR among different groups.

The structure of gut microbiota is critical to intestinal function (David et al. 2014). The composition of gut microbes is influenced by factors such as antibiotic treatment and diet (Clemente et al. 2018; Dixit et al. 2021). At 14 and 21 days, 0.1% and 0.2% β-1,3/1,6-glucan supplementation increased cecal microflora diversity, respectively. At 28 days, supplementation of 0.1% and 0.2% β-1,3/1,6-glucan increased cecal microflora diversity, but no significant difference. The results showed that different glucan contents had different effects on the gut microbiota diversity of broilers at different ages. Fassarella et al. found that β-glucan had no significant difference in the alpha diversity of gut microbiota in early pigs, and a PCoA analysis showed that β-glucan had a significant effect on feces samples before weaning (Hugo et al. 2020). Velikonja et al. showed that different daily intakes of glucan (6 g vs. 3 g per day) had different effects on gut microbiota diversity (Velikonja et al. 2019). These results are similar to our findings. The addition of different glucan levels (0.1% and 0.2%) has different effects on the gut microbiota diversity of chicks. However, our study discovers that adding different levels of β-1,3/1,6-glucan may have the opposite effect on the diversity of gut microbiota.

The interaction between diet-microorganism-host is closely related to body health and disease (Clemente et al. 2018). Dysbiosis of the gut microbiota can lead to diseases as diverse as inflammatory bowel disease (IBD), systemic inflammatory arthritis, and multiple sclerosis (Clemente et al. 2018; Velikonja et al. 2019). In this study, the dominant bacteria in the cecal contents of chicks were *Alistipes*, *Bacteroides*, *Ruminococcus*_torques_group, unclassified_f__*Lachnospiraceae*, *Faecalibacterium* and *Lactobacillus*. Studies have shown that *Alistipes* produce short-chain fatty acids and reduce intestinal inflammation, which may have a protective effect on many diseases, including liver fibrosis, colitis, cancer immunotherapy, and cardiovascular disease (Parker et al. 2020). *Bacteroidetes* and *Faecalibacterium* have been positively correlated with human health and are considered health-promoting gut microbiota (Wang et al. 2020). *Bacteroides* encodes polysaccharide binding proteins in the outer membrane through polysaccharide utilization sites (PUL) to capture polysaccharides and decompose polysaccharides into oligosaccharides to promote the utilization and uptake of dietary polysaccharides by *Bacteroides* (Tamura et al. 2017). *Faecalibacterium* facilitates the utilization of acetate in the intestine (Virginio Junior et al. 2021). Acetate is used as an energy source for the liver and peripheral tissues and as a signal molecule in gluconeogenesis and lipogenesis (Zambell et al. 2003). *Ruminococcus*_torques_group and unclassified_f__*Lachnospiraceae* belong to Firmicutes (Bassanini et al. 2019). *Ruminococcus*_torques aggravates the symptoms of neurodegenerative disease amyotrophic lateral sclerosis (Blacher et al. 2019). Mucosa-associated bacteria *Ruminococcus*_torques are significantly increased in the intestinal epithelial cells of IBD, such as ulcerative colitis and Crohn’s disease (Png et al. 2010). Although members of *Lachnospiraceae* are among the primary producers of short-chain fatty acids, the different taxonomic groups of *Lachnospiraceae* have also been associated with different enteral and parenteral diseases (Vacca et al. 2020). Metabolic syndrome, obesity, diabetes, liver disease, and IBD are all inflammatory diseases associated with the *Lachnospiraceae* family or specific taxonomic groups of *Lachnospiraceae* (Vacca et al. 2020; Zeng et al. 2016). In addition, they appear to be associated with major depressive disorder and multiple sclerosis syndrome (Cheung et al. 2019; Vacca et al. 2020). Meanwhile, the increased abundance of *Lachnospiraceae* was associated with aging (Odamaki et al. 2016).

This study found that adding 0.2% β-1,3/1,6-glucan to the diet of 14 days of age yellow-feathered broilers can significantly improve the abundance of *Faecalibacterium* that promote intestinal health and significantly reduce the abundance of unclassified_f__*Lachnospiraceae* associated with host aging and many diseases. Supplementation of 0.1% β-1,3/1,6-glucan in diets of yellow-feathered broilers at 21 days of age significantly reduced the abundance of *Ruminococcus*_torques_group, which is associated with host enteritis and neurological diseases. Supplementation of 0.1% β-1,3/1,6-glucan at 28 days of age significantly increased the abundance of *Alistipes*, which promotes intestinal nutrition and protection. Huali et al. reported that β-glucan significantly increased the abundance of *Bacteroidetes* and *Faecalibacterium*. On the contrary, the abundance of *Lachnospiraceae* and *Ruminococcus* significantly decreased, similar to the results in this study (Wang et al. 2020). Hugo et al. found significant differences in *Ruminococcus* in feces when β-glucan dietary intervention was administered at the pre-weaning stage, highlighting the potential regulatory role of the microbiota on dietary fiber (Hugo et al. 2020). Angelis et al. reported that after the intervention of a barley glucan diet on the human body, the abundance of *Ruminococcus* increased, and the abundance of other *Firmicutes* such as *Faecalibacterium* decreased (De Angelis et al. 2015). These results indicate that dietary supplementation of β-glucan can regulate the gut microbiota structure, and the source and amount of β-glucan can significantly affect sensitive bacteria. In this study, the supplementation of 0.1% and 0.2% β-1,3/1,6-glucan in diets of yellow-feathered broilers had different effects on the cecal microflora structure of broilers at different ages. Supplementation of 0.2%, 0.1%, and 0.1% β-1,3/1,6-glucan in the diets of yellow-feathered broilers at 14, 21, and 28 days of age had significant effects on the maintenance of gut microbiota structure that was more conducive to intestinal nutrient absorption and immune resistance.

In this study, we found that yellow-feathered broilers from hatching to the 28th day were highly AMR to 9 commonly used antibiotics. At 14, 21, and 28 days of age, yellow-feathered broilers supplemented with 0.2%, 0.1%, and 0.1% β-1,3/1,6-glucan significantly changed the gut microbiota composition and beneficial bacteria such a beneficial bacteria *Alistipes*, *Bacteroides* and *Faecalibacterium* significantly increased. These results demonstrate that β-1,3/1,6-glucan as an antibiotic substitute can improve gut microbiota composition, which is helpful for the promotion and application of alternative antibiotic products.

## Data Availability

The data presented in this study are deposited in the (NCBI SRA) repository (accession number: PRJNA820361).
